# The relationship between health mindsets and health protective behaviors: An exploratory investigation in a convenience sample of American Indian adults during the COVID-19 pandemic

**DOI:** 10.1371/journal.pone.0242902

**Published:** 2020-11-30

**Authors:** Neha A. John-Henderson, Claudia M. Mueller

**Affiliations:** 1 Department of Psychology, Montana State University, Bozeman, Montana, United States of America; 2 Department of Surgery, Stanford University School of Medicine, Stanford, California, United States of America; CentERdata, NETHERLANDS

## Abstract

The Novel-coronavirus disease-2019 (COVID-2019) outbreak was declared a national emergency on March 13, 2020. To reduce the spread of the virus, Americans were asked to physically distance and to increase disinfecting behaviors such as hand washing. Previous research indicates that one’s mindset about health, or the degree to which they view health as fixed or modifiable, influences health behaviors. Current data indicates that American Indians (AIs) are at greater risk for COVID-19. As such, it is important to understand whether mindsets about health may affect behaviors which could prevent spread of the virus in AIs. In this exploratory investigation, a convenience sample of two hundred AI adults completed a questionnaire one month prior to the declaration of the COVID-19 pandemic as a national emergency. They provided demographic information and completed a measure of health mindsets. The second wave of data was collected approximately one month later, where we collected a measure of physical distancing behavior and a measure of disinfecting behaviors. In AI adults, health mindset predicted frequency of physical distancing behaviors and disinfecting behaviors, with individuals who viewed health as less fixed engaging in more physical distancing and disinfecting behaviors, while individuals who viewed health as more fixed reported less physical distancing and less disinfecting behaviors. In AIs, growth health mindsets predicted physical distancing and disinfecting behaviors, both of which are important in reducing the spread of COVID-19. Interventions which are designed to promote growth mindsets of health may promote health-protective behaviors in the context of the COVID-19 pandemic.

## Introduction

American Indians (AIs) are disproportionately affected by chronic physical health diseases, including cardiovascular disease, diabetes, and obesity [1]. These conditions are risk factors for the novel Corona Virus (COVID-19). This appears to in part explain why thus far, AIs have been disproportionately affected by the virus [2–5]. Since the COVID-19 pandemic was declared a national emergency on March 13, 2020, recommendations were made to assist in reducing the spread of the virus. These recommendations included physical distancing, or keeping 6 feet of distance from other people and avoiding large groups or crowded places [6, 7]. Furthermore, the CDC recommended increasing frequency of handwashing, use of hand sanitizer and disinfecting wipes as appropriate [8]. Given that AIs are an at-risk population, it is important to identify psychological factors which increase the likelihood of following these recommendations.

One potential factor is mindset. A “mindset” is a group of beliefs or assumptions that individuals hold which can influence their behaviors and perceptions [9]. Mindsets can be divided into two categories: fixed and growth. Differences in mindset can affect the way in which different individuals respond to the same circumstances. A fixed mindset is characterized by the belief that certain attributes are not modifiable, whereas a growth mindset is characterized by the belief that an attribute can be changed by behaviors and actions [9]. A substantial body of work highlights the importance of mindset in shaping stress responses [10–12], educational outcomes [13–16], and personal relationships [17, 18]. More recently, mindsets have been studied in the context of health. This growing area of research indicates that mindsets about health-relevant outcomes (e.g. weight and fitness) affect motivation and engagement in health behaviors [19, 20]. Individuals may also hold mindsets about health as a broad construct. Some individuals may believe that health is fixed or predetermined (i.e. “fixed” health mindset), while others may believe that health is modifiable or can be shaped by behavioral changes (i.e. “growth” health mindset). In our previous work in American Indian (AI) college students, growth mindsets specific to health predicted lower body mass index, a relationship which was in part explained by higher levels of physical activity as measured by wrist-accelerometry [21]. These findings suggest that holding a growth health mindset may encourage engagement in behaviors which are health-protective.

As the COVID-19 pandemic continues to threaten the health of individuals across the world, it is increasingly important to identify factors which may promote relevant preventative behaviors such as physical distancing and disinfecting behaviors. This knowledge may be particularly important for at-risk individuals and communities such as AIs, who are disproportionately affected by COVID-19 compared to other racial and ethnic groups [2–5]. To date, it is unknown whether one’s mindset about health (i.e. fixed vs. growth) predicts the degree to which individuals engage in social distancing. If a growth health mindset associates with greater engagement in physical distancing and disinfecting behaviors, it could be a psychological factor which would reduce the spread of COVID-19 in AI communities.

Here, in a sample of 200 American Indian adults, we investigate i) whether health mindsets (measured before the pandemic) predict engagement in physical distancing and disinfecting behaviors. We hypothesized that a more pronounced growth health mindset would predict greater physical distancing behavior and greater frequency of disinfecting behaviors.

## Methods

This study was approved by the Montana State University Institutional Review Board (Approval number: NJ-H042220-EX). Participants for the current research were drawn from a previous cross-sectional survey focused on the identification of psychosocial predictors of health and well-being in older AI adults. The participants were originally recruited by Qualtrics using convenience sampling. Qualtrics draws participants from multiple managed research panels. For hard to reach groups, including AIs, Qualtrics uses niche panels brought about through specialized recruitment campaigns. To be eligible to participate in the Qualtrics cross-sectional survey, participants had to identify as American Indian, currently live within the United States, and be at least 30 years old. In this investigation, participants provided their email addresses to allow for participation in possible follow-up studies. We contacted all 300 of these participants with the hope of obtaining a sample of 200 AI adults. The sample size of 200 was determined based on minimum recommendations for the data analysis plan associated with a larger longitudinal project focused on tracking changes in health and well-being in older AI adults. Specifically, a minimum sample of 200 was estimated to provide sufficient power for longitudinal structural equation modeling for the ongoing longitudinal research [22]. Sensitivity analyses were performed to identify the minimum effect sizes detectable with 200 participants used in this study. Based on Monte Carlo simulations (N = 10,000), 200 participants provided sufficient power (>80%) to detect a minimum effect of (β = .15) assuming α = .05, which is consistent with our past research using similar construct. Thus, we are confident that the current sample size provides sufficient power to test our research questions.

Out of the 300 participants from our past work, we had a sample of 210 interested AI adults who formed an online panel. There were no statistically significant differences in age, income, or health mindset between those who chose to participate in this study and those who did not. All participants provided written informed consent before participation. Time 1 data was collected during the final week of February, 2020, prior to the declaration of the COVID-19 pandemic. The second wave of data was collected during the final week of April and the first week of May, 2020. Two hundred of the 210 participants from Time 1 completed data collection at Time 2. There were no statistically significant differences in demographics or health mindsets between the initial sample of 210 and the 200 participants who completed Time 2 data collection. The analyses we report here are based on the 200 participants for which we had complete data. Participants were given $10 Amazon gift cards for their completion of each survey. To be eligible for participation, participants had to self-identify as American Indian and be over the age of 18. Surveys administered at Time 1 included demographic questions and a measure of health mindset. Several other measures were administered at Time 1 which are not relevant to the current report including measures of historical loss and other health behaviors. At the second wave of data collection, we measured the degree to which participants engaged in physical distancing and the frequency of disinfecting behaviors.

### Measures

#### Health mindset

A three-item Health Mindset Scale was developed based on established mindset questionnaires created by Carol Dweck and her colleagues which has been previously applied in domains such as intelligence and moral character [17]. In this research this measure was our independent variable. Participants are instructed to report their agreement with each of the following statements on a six-point Likert scale (1—strongly agree, 6—strongly disagree): “Your body has a certain amount of health, and you really can’t do much to change it,” “Your health is something about you that you can’t change very much,” and “You can try to make yourself feel better, but you can’t really change your basic health.” (See S1 File). Consistent with recent mindset research, health mindset was modeled as a continuous variable in our analyses [21]. Lower numbers reflect a low growth health mindset, and higher numbers reflect a high growth health mindset. We have used this scale previously in a sample of AIs [21]. The scale demonstrated good internal reliability in this sample (α = .91).

#### Physical distancing behaviors

One of the dependent variables in this research was the frequency with which AI adults physically distanced. We defined physical distancing as keeping at least 6 feet away from other people, and limiting time spent in crowded places or in groups [7]. We then asked participants, based on this definition of physical distancing, in the past 7 days, to what extent did you engage in physical distancing? Participants responded using a 5-point scale including 1(*not at all*), 2 (*a little*), 3 (*somewhat*), 4(*a lot*) and 5(*a great deal*). This scale has been used previously as an index of engaging in social distancing [23]. We changed the wording of the question to be physical distancing instead of social distancing in line with the World Health Organization.

#### Disinfecting behaviors

Our second dependent variable was the self-reported frequency of disinfecting behaviors. We measured the frequency with which participants engaged in disinfecting behaviors using 4 items (α = 0.90). These 4 items measured the frequency with which participants washed their hands, used hand sanitizer, cleaned their phones and used disinfecting wipes in the last 7 days using a 5- point scale ranging from not at all (1 point) to very often (5 points) [24].

#### Covariates

Self-reported age, self-reported biological sex, and self-reported annual income were collected at Time 1 and were used as covariates in our statistical analyses. Annual income was reported on a scale from 1 (below US$20,000), 2(US$20,000-$40,000), 3(US$40,001–60,000), 4(US$60,001–80,000), 5(US$80,001–100,000) and 6 (US$100,001 and above) [25, 26].

### Data analyses

Statistical Analyses were conducted using SPSS (Version 24; IBM, Armonk, NY). Continuous covariates were centered with *z-*scores prior to being used in analyses. Participant self-reported biological sex was coded as 1 = female, 2 = male. Initial Pearson product-moment correlation analyses were performed to determine bivariate associations between demographic variables, health mindsets, physical distancing behaviors, and disinfecting behaviors at Time 2. Next, we used hierarchical linear regressions controlling for age, biological sex and income to test whether health mindsets before the onset of the COVID-19 pandemic predicted physical distancing and disinfecting behaviors. All data used in the analyses are included in S1 Dataset.

## Results

Our sample consisted of 200 AI adults (mean age = 55.09, SD = 13.10). 59.0% identified as female, and 40.5% identified as male. Only 7.4% of our sample lived on a tribal reservation at the time of the survey. Approximately half of the participants indicated that they were enrolled members of a tribe (48.3%). Table 1 presents descriptive statistics and Table 2 presents bivariate correlations between main variables of interest.

**Table 1:** Descriptive statistics.

	Range	Mean	Standard Deviation
Age (Time 1)	30–99	55.09	13.10
Annual Income (Time 1)	1–6	2.96	1.72
Health Mindset (Time 1)	3–18	10.25	3.65
Physical Distancing (Time 2)	1–5	2.81	1.34
Disinfecting behaviors Time 2)	1–5	2.56	1.03

Note: The annual income scale was categorized as follows: 1: (below US $20,000), 2: (US $20,001-$40,000), 3: (US$ 40,001-$60,000), 4: (US$ 60,001-$80,000), 5: (US $80,001-$100,000), and 6: (US$ 100,001 and above).

**Table 2:** Bivariate correlations between main variables of interest.

Variable	1	2	3	4	5	6
1. Age (time 1)	-					
2. Sex (time 1)	.08	-				
3. Income (time 1)	.07	.31**	-			
4. Health Mindset (time 1)	-.04	.05	-.03	-		
5. Physical distancing frequency (time 2)	-.15*	-.02	-.14	.67**	-	
6. Disinfecting Behaviors (time 2)	.05	-.07	.004	.72**	.52**	-

Note:

*Correlation significant at the .05 level (two-tailed)

** Correlation significant at the .01 level (two tailed)

Using a linear hierarchical regression model controlling for age, sex and annual income, we found that health mindset predicted the frequency with which participants engaged in physical distancing practices one month after the onset of the pandemic (β = .66 t = 12.75, *p* < .001, ΔR^2^ = .44), with growth health mindsets predicting more physical distancing. In a separate hierarchical regression model with the same covariates, health mindset predicted disinfecting behaviors (β = .73, t = 15.05, *p* < .001, ΔR^2^ = .53), with growth health mindsets predicting more frequent disinfecting behaviors (See Table 3).

**Table 3:** Hierarchical linear regression model with Health Mindset predicting physical distancing (3a) and disinfecting behaviors (3b).

3a)	β	*p*	ΔR^2^	Lower CI	Upper CI
**Step 1**					
Age	-0.11	.04		-0.02	-0.001
Biological Sex	-0.01	.88		-0.31	0.27
Annual Income	-0.11	.05		-0.17	-0.002
**Step 2**					
Health Mindset	0.66	.000	.44	.21	.28
3b)	β	*p*	ΔR^2^	Lower CI	Upper CI
**Step 1**					
Age	0.09	.08		-0.001	0.01
Biological Sex	-0.13	.01		-0.47	0.06
Annual Income	0.06	.25		-0.03	0.10
**Step 2**					
Health Mindset	0.73	.000	.53	.18	.23

## Discussion

After the declaration of the COVID-19 pandemic as a national emergency in the United States, physical distancing recommendations were issued in order to reduce the spread of the virus. Here, we were interested in whether in AIs, who are at increased risk for COVID-19 [2–5], one’s health mindset would predict the degree to which they engaged in physical distancing and disinfecting behaviors. Specifically, we hypothesized that AI adults who viewed health as strongly modifiable by behavior (i.e. high growth health mindset) would be more likely to engage in physical distancing behaviors and disinfecting behaviors. This hypothesis was based on previous research indicating that a growth health mindset predicts greater levels of engagement in behaviors that are health protective [19–21]. Specific to COVID-19, it is possible that holding a growth mindset about health shapes a belief that risk of infection is not pre-determined or fixed and can be minimized by protective behaviors. Our findings supported our hypothesis, raising the possibility that brief interventions targeting mindsets about health may promote physical distancing and disinfecting behaviors in the context of the COVID-19 pandemic.

There are important limitations to note. First, all data was collected online, thus participants had to have access to a computer and internet in order to participate. As a result, the population of AIs who do not have this access are not represented. However, approximately 50% of our participants made less than $40,000 annually, indicating that our sample includes AIs with relatively low annual incomes. Our data also is unable to speak to potential differences that may be specific to residing in urban settings compare to residing on tribal reservations. It is possible that physical distancing and disinfecting behavior patterns and the implications of these behaviors may be different in these different living environments. Thus far, our focus has been on physical distancing and disinfecting behaviors and we therefore do not know whether mindset might also influence the adoption of other protective health measures, such as mask-wearing. Additional investigations are needed to understand the broader effect of health mindset on other health-protective behaviors. In the current research, we did not have a measure of occupation. It is possible that certain occupations inherently limit the possibility of physical distancing. For example, individuals working in health care settings may not be able to physically distance from patients. This is a limitation on the internal validity of the findings, and future work should measure occupation to better account for how occupational demands and environments may affect the observed relationships. Finally, the overarching aim of the research study from which this sample was drawn from was to identify psychosocial correlates of health and well-being in older AI adults. As a result, the current sample was limited to AI adults over the age of 30. To improve generalizability, future work should investigate whether the relationship observed here between health mindsets and health-protective behaviors is similar in younger AI adults.

Mindset interventions, such as those employed effectively in school settings and interpersonal relationships, could be applied to behavior modification during the COVID-19 crisis. For instance, Yeager and Dweck and their colleagues were able to demonstrate consistent improvement in grades and challenge-seeking behaviors for students after a simple one-hour, online growth-mindset intervention that emphasized the malleability of learning [15]. Yeager was also able to alter responses to bullying in teenagers with interventions that targeted mindsets of behavior change [27].

Generally, successful growth mindset interventions have been brief and scalable and explicitly link behavior and effort to the improvement of abilities. For example, teaching children that “the brain is a muscle” which gets stronger through use can lead to improved academic outcomes for students from grade school to university [15, 16]. Similarly, growth-mindset messages which focus on strengthening health through behavior such as “you protect your health when you distance and disinfect” or “you protect yourself from viruses when you wash your hands and wear a mask” might have the same positive effect on target audiences within public health.

Prior research indicates that it may be important to tailor the focus and content of interventions to fit specific features of the target audience. One important audience feature is participant age as developmental changes in cognitive processes may influence how mindset interventions are perceived. Specifically, young adolescents are concrete thinkers who may struggle to comprehend abstract concepts such as health and who may have difficulty understanding how their behaviors can affect future outcomes [28]. However, in later adolescence abstract thinking becomes more accessible, allowing individuals to understand how their current behaviors may affect their future health [28, 29]. In line with this, it is possible that health mindset interventions directed at younger audiences to change behavior during the ongoing COVID-19 pandemic should focus on concrete actions with immediate implications such as handwashing or sanitizing to decrease viral spread. In contrast, health mindset interventions targeting older populations may focus on more complex or abstract concepts such as the adaptation of social behaviors and daily routines to protect individual and societal health during the COVID pandemic.

Mindset is a relatively new concept within health but one which has shown initial promise [30–33]. Our findings suggest that holding a growth mindset of health can be linked to greater adoption of behaviors which may work to reduce the transmission of COVID-19 in vulnerable communities. We believe that these findings add to our growing understanding of human behavioral responses to the COVID-19 pandemic and may lead the development of more adaptive responses to this health crisis. Future investigations could focus on how mindset may influence other COVID-related health protective behaviors such as mask and glove wearing both within the general population and in specific subgroups. In addition, it could be interesting to examine how mindset might differentially affect behavior for certain populations such as the elderly or individuals with other underlying vulnerability to disease. Finally, the development and testing of specific pandemic-related mindset interventions may prove to be valuable additions to public health initiatives.

## Supporting information

S1 File Health mindset scale. (DOCX)

S1 Dataset SPSS file including data for all variables included in the reported analyses. (SAV)
