# Supplementary material for: The relationship between health mindsets and health protective behaviors: An exploratory investigation in a convenience sample of American Indian adults during the COVID-19 pandemic
Source: PLoS One. 2020 Nov 30;15(11):e0242902. doi: 10.1371/journal.pone.0242902 (PMC7703932; doi:10.1371/journal.pone.0242902)
Supplement: S1 File — (DOCX) [file pone.0242902.s001.docx]

# Health Mindset Scale:

# Please indicate how much you agree with the following statements:

# 1) Your body has a certain amount of health, and you really can’t do much to change it:

# Strongly Strongly

# Agree Disagree

# 1 2 3 4 5 6

# 2) Your health is something about you that you can’t change very much:

# Strongly Strongly

# Agree Disagree

# 1 2 3 4 5 6

# 3) You can try to make yourself feel better, but you can’t really change your basic health.

# Strongly Strongly

# Agree Disagree

# 1 2 3 4 5 6
